# Changes in employment psychology of Chinese university students during the two stages of COVID-19 control and their impacts on their employment intentions

**DOI:** 10.3389/fpsyg.2024.1447103

**Published:** 2024-11-25

**Authors:** Sining Zheng, Yanfen Yan

**Affiliations:** School of Public Administration and Law, Fujian Agriculture and Forestry University, Fuzhou, China

**Keywords:** COVID-19 pandemic, university students, mental health, employment psychology, China

## Abstract

**Introduction:**

The employment psychology and issues being faced by university students have garnered significant attention after the COVID-19 outbreak.

**Methods:**

Focusing on Chinese university students, this study compares the changes in their employment psychology between the initial and normalization stages of COVID-19 prevention and control in China and examines their employment choices during the normalization stage. This study also investigates how the transformed employment psychology of these students influences their employment intentions.

**Results:**

(1) Chinese university students exhibit stronger feelings of employment confusion, anxiety, stability preference, uneasiness, and inferiority during the normalization stage compared with the initial stage, thus leading to a more negative employment psychology; (2) during the normalization stage, university students display a more pessimistic outlook on their employment situation and show their preference for direct employment, particularly in public institutions, state-owned enterprises, and government organizations. They also show a greater interest in working in first-tier cities than in their hometowns; (3) the employment psychology of these students in the post-pandemic period significantly influences their employment intentions, with employment stability preference psychology playing a crucial role; and (4) the employment stability preference psychology positively and significantly affects the employment intentions of junior college students. Conversely, this employment stability preference psychology has a negative effect on the employment intentions of academic master’s and doctoral students. This paper also proposes some strategies to enhance the employment psychology of university students and improve their quality of employment at various levels, including government, school, enterprise, family, and individual.

## 1 Introduction

In December 2022, the State Council released the Notice on Further Optimizing the Implementation of COVID-19 Prevention and Control Measures, signifying China’s transition to a normalized stage of control efforts. As a global public health emergency, The COVID-19 pandemic brought significant and widespread risks with a “social amplification” effect. Apart from its visible material impacts, the pandemic also resulted in enduring “trauma” at deeper levels of social psychology and cultural spirituality (Kasperson et al., 2022). During the period of COVID-19 prevention and control in China, university students were required to accept campus lockdowns or study online at home, which negatively affected their mental health (Xu et al., 2023). As a result, these students became prone to negative mentalities, such as anxiety, depression, and confusion (Kumar et al., 2020; Sae et al., 2022), that tend to linger for an extended period.

The Ministry of Education projects 11.79 million students to graduate from universities in 2024, thus imposing an unprecedented employment pressure on these students. Given the new stage of pandemic control and the challenging employment landscape in China, what shifts have taken place in the employment psychology of university students during the two stages of COVID-19 prevention and control? What are their employment choices? How does their employment psychology impact their employment intentions? By answering these questions, this study carries significant theoretical and practical implications.

This study thus compares the employment psychology of Chinese university students between the initial and normalization stages of COVID-19 prevention and control in China, examines their employment choices during the normalization stage, and investigates how their transformed employment psychology influences their employment intentions.

## 2 Literature review

The COVID-19 pandemic was a collective traumatic event that negatively impacted the mental health and spiritual wellbeing of individuals. Developmental contextualism suggests that an individual’s emotional states are influenced by a combination of environmental characteristics and individual factors at a given point in time (Lerner, 2002; Lerner, 2006). Many studies have shown significant increases in anxiety, depression and stress levels, as well as increased sleep disturbance, social isolation and post-traumatic stress disorder among individuals of all ages from different countries as a result of COVID-19 pandemic (Brooks et al., 2020; Regnoli et al., 2022; Kira et al., 2021; Rodolfo et al., 2023; Kelsey et al., 2024). The environmental characteristics of COVID-19 pandemic, such as its sudden onset and harmful effects, when interacting with immature university students can have a clear impact on their emotional and psychological wellbeing. Many studies have also investigated the mental health status of university students under the influence of the pandemic. Some scholars compared the mental health status of these students before and after the pandemic and found that these students experienced increased loneliness, psychological distress, suicidal ideation and decreased euphoria and resilience (Park and Khanh, 2023). Other scholars found that the mental health problems among these students significantly increased during the pandemic, which was accompanied by deepened levels of depression, anxiety, and stress (Priya and Kakoli, 2023).

Some studies also investigated those factors that influence these university students’ mental health status, with several researchers arguing that such status is contingent upon individuals’ awareness of the pandemic. In other words, having more knowledge about COVID-19 prevention and control measures increases the confidence of these students in overcoming the pandemic and subsequently improves their mental health (Wang et al., 2022). To prevent and control the COVID-19 pandemic, the university adopted an online teaching model, which was later changed to a hybrid teaching model combining online and offline. The change in teaching model may have a negative impact on students’ mental health. Online learning may change students’ attitudes and engagement, leading to a decrease in their learning outcomes (Yi, 2024). Such negative effects also include poorer physical health, rebellious and depressive moods, and concerns about future employment among students (Zhang and Bian, 2024). Employment, academic, and economic pressure are the largest psychological stressors for university students during the pandemic, followed by interpersonal and emotional pressures (Zhang, 2023). Physical condition is another risk factor that leads to the negative psychology of university students; thus schools should pay attention to the physical and psychological health of their graduates (Li J. et al., 2021).

Previous scholars have also investigated the employment psychology of university students under the influence of COVID-19 pandemic, with some revealing that the pandemic has brought various degrees of employment psychology problems among these students, including comparison and herd mentality in the cognitive aspect, anxiety and depression in the emotional aspect, and avoidance, confusion, and inaction in the behavioral aspect (Zhou et al., 2023; Yu and Cui, 2021). Griffin explored the psychological impact of working during the COVID-19 pandemic on medical and nursing students, showing that work has had a significant negative impact on students’ psychological wellbeing, as a result of demanding working conditions, unprecedented exposure to death and suffering and lack of preparation for new job roles (Griffin and Riley, 2022).

Other studies noted that changes in employment psychology due to the pandemic also influenced these graduates’ employment choices. Regarding employment units and locations, some scholars found that after the COVID-19 pandemic, university graduates demonstrated a strong employment risk aversion, thus driving them to join the public sector; they also reported a significant increase in the number of graduates choosing to work in government organizations and in areas rich in medical resources (Huang, 2020; Li, 2023a). The employment cognition of university graduates tends to be negative, and their recognition of private enterprises is low (Ye and Chen, 2023). In terms of expected salary, some scholars found that during the COVID-19 prevention and control stages, the employment anxiety of university graduates intensified, thus forming a highly rational and objective employment behavior and driving their willingness to lower their salary and work location expectations to adapt to the job market (Zhang et al., 2022). However, some scholars contradicted these claims and argued that due to the long COVID-19 lockdowns, university students felt some “material scarcity” to a certain extent, which made them realize the importance of salary when job hunting (Li X. et al., 2021). Certain measures, such as employment guidance, can be used to alleviate the pandemic anxiety of university students and enhance their confidence in employment (Zheng et al., 2022). Previous studies on the employment of university students under the impact of COVID-19 pandemic have mainly focused on these students’ outlook on employment, employability, quality of employment, willingness to return to their hometowns for employment, and choice of employment units and locations (Li, 2020a; Feng et al., 2022).

Scholars at home and abroad have made fruitful research contributions to the impact of the COVID-19 on the mental health of university students, and some studies have also explored the employment psychology and employment behavior of these students. However, several issues have been neglected in the literature. First, while scholars have comparatively analyzed the mental health status of university students before, during, and after the outbreak of the COVID-19 pandemic, only a few studies have investigated the changes in the employment psychology of these students between the initial and normalization stages of COVID-19 prevention and control, especially in the Chinese context. Second, most scholars have focused on the employment psychology of university graduates and ignored the employment psychology and behavior of university students of different academic levels and grades. Third, previous studies on the influence of university students’ employment psychology on their employment choices are mostly theoretical, and only few studies have empirically investigated the influence of university students’ post-pandemic employment psychology on their future employment intentions.

## 3 Changes in employment psychology of university students during the two stages of COVID-19 prevention and control

### 3.1 Data sources and sample description

In December 2022, the State Council released the Notice on Further Optimizing the Implementation of COVID-19 Prevention and Control Measures and the Overall Plan on the Implementation of ‘Class B’ for COVID-19, which marked a new stage of pandemic control in China.

The data used in this paper comes from the survey questionnaire distributed by the research group from September to December 2023. The participants were current Chinese university students, including junior college, undergraduate, professional master’s, academic master’s and doctoral students. In addition, participants were required to have experienced at least 1 year of campus COVID-19 prevention and control. Therefore, we excluded the first year junior college and first year undergraduate students to ensure that all participants in the sample had experienced different stages of COVID-19 prevention and control to truly reflect the changes in students’ employment psychology in the two stages of COVID-19 prevention and control.

**Figure 1:**
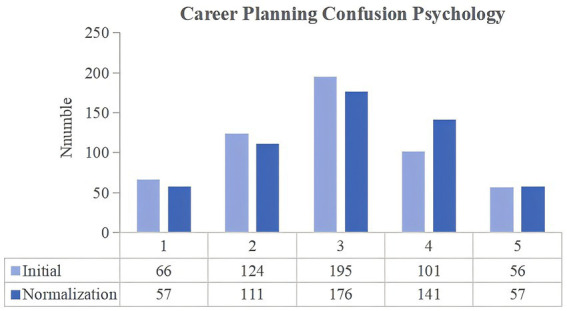
Changes in the career planning confusion psychology of university students between the two stages of COVID-19 pandemic control.

**Figure 2:**
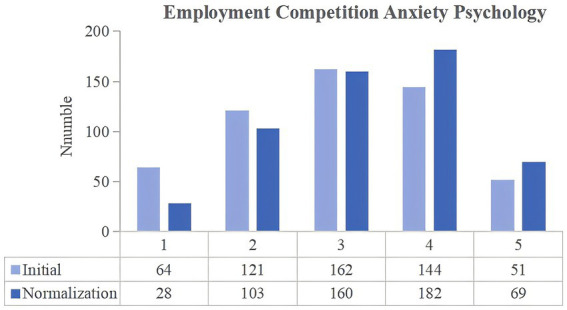
Changes in the employment competition anxiety psychology of university students between the two stages of COVID-19 pandemic control.

**Figure 3:**
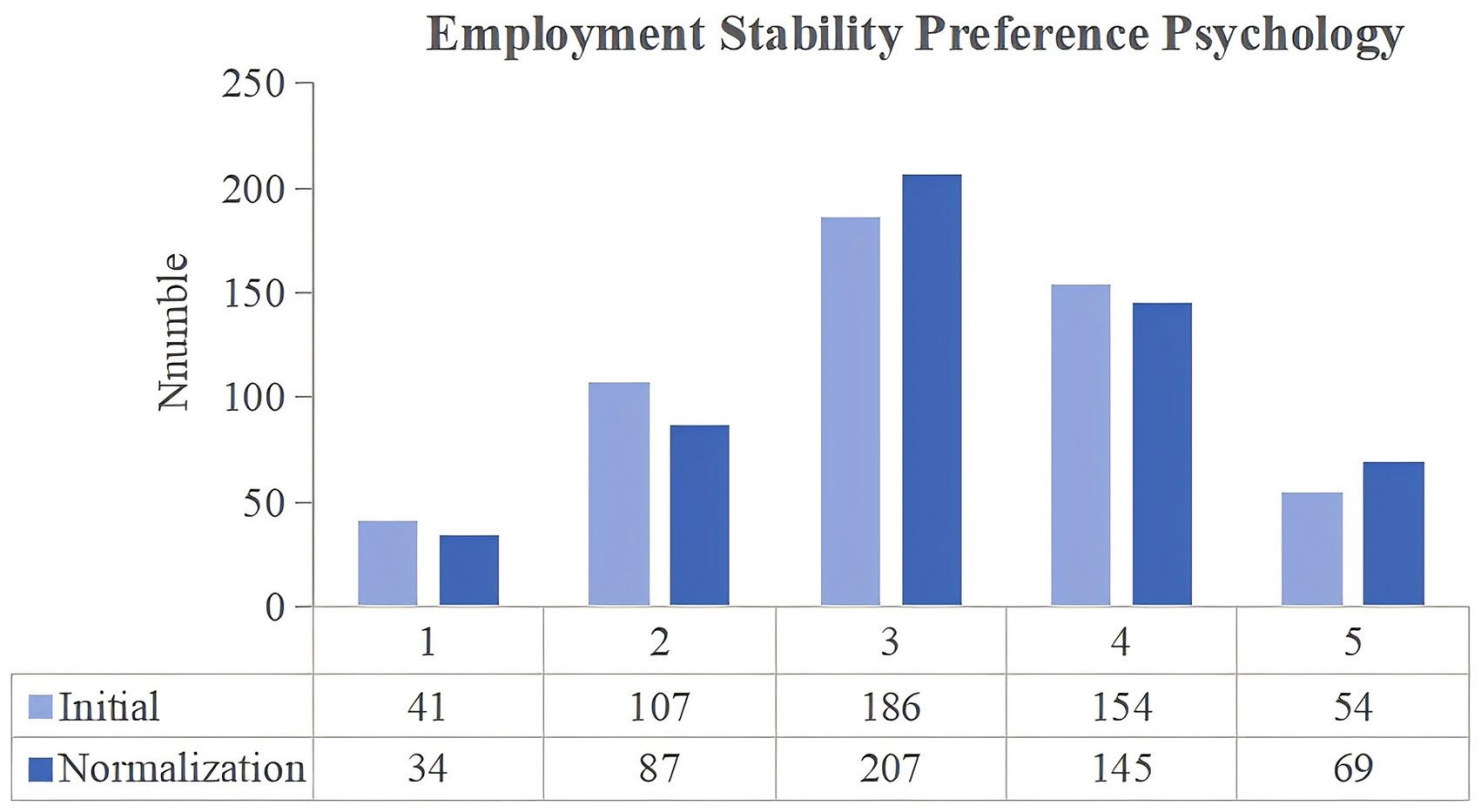
Changes in the employment stability preference psychology of university students between the two stages of COVID-19 pandemic control.

**Figure 4:**
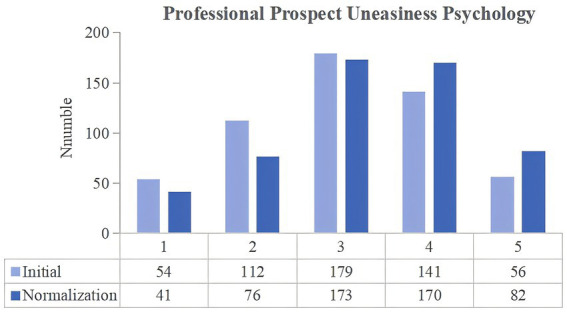
Changes in the professional prospect uneasiness psychology of university students between the two stages of COVID-19 pandemic control.

**Figure 5:**
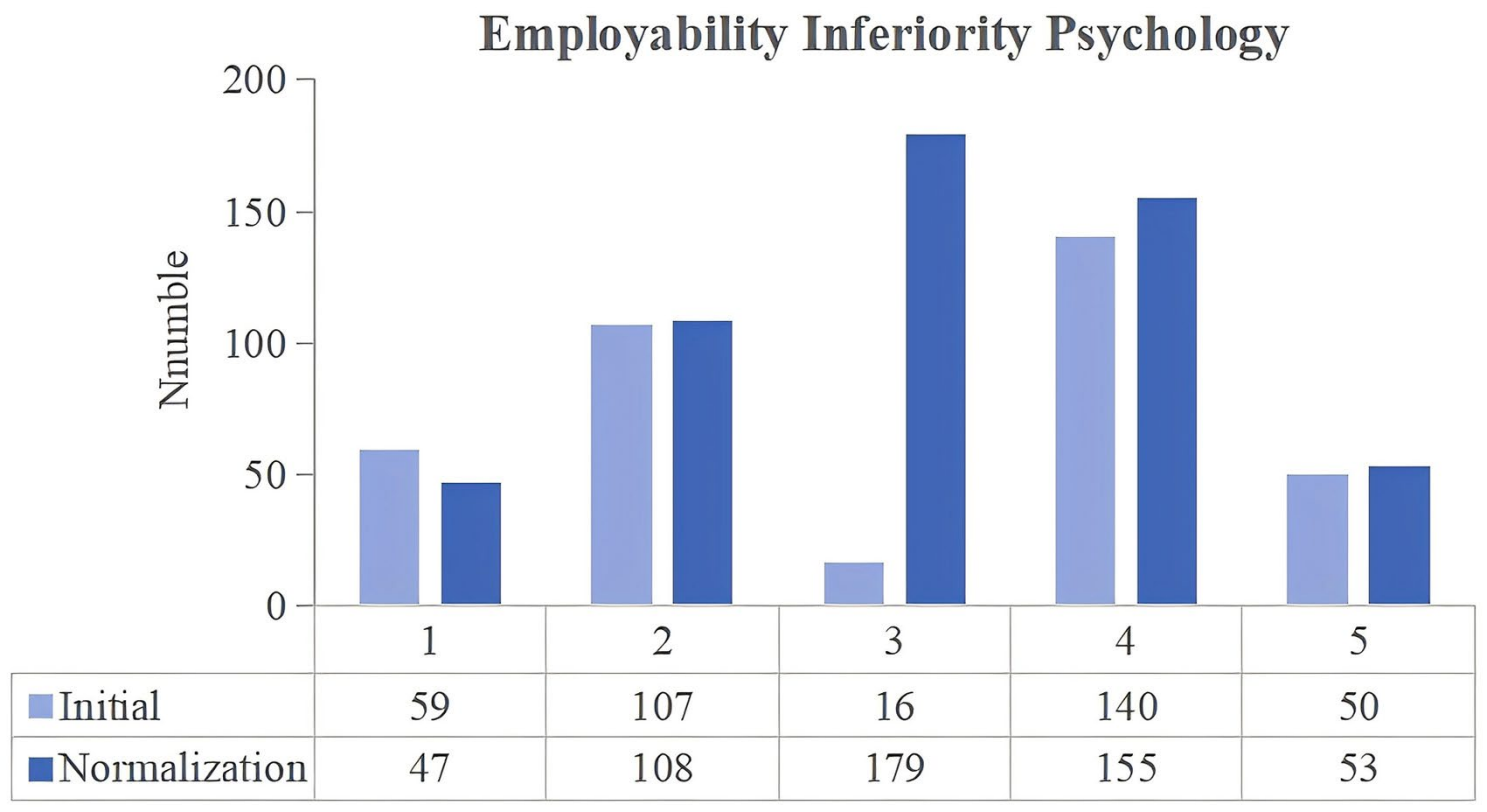
Changes in the employability inferiority psychology of university students between the two stages of COVID-19 pandemic control.

**Figure 6:**
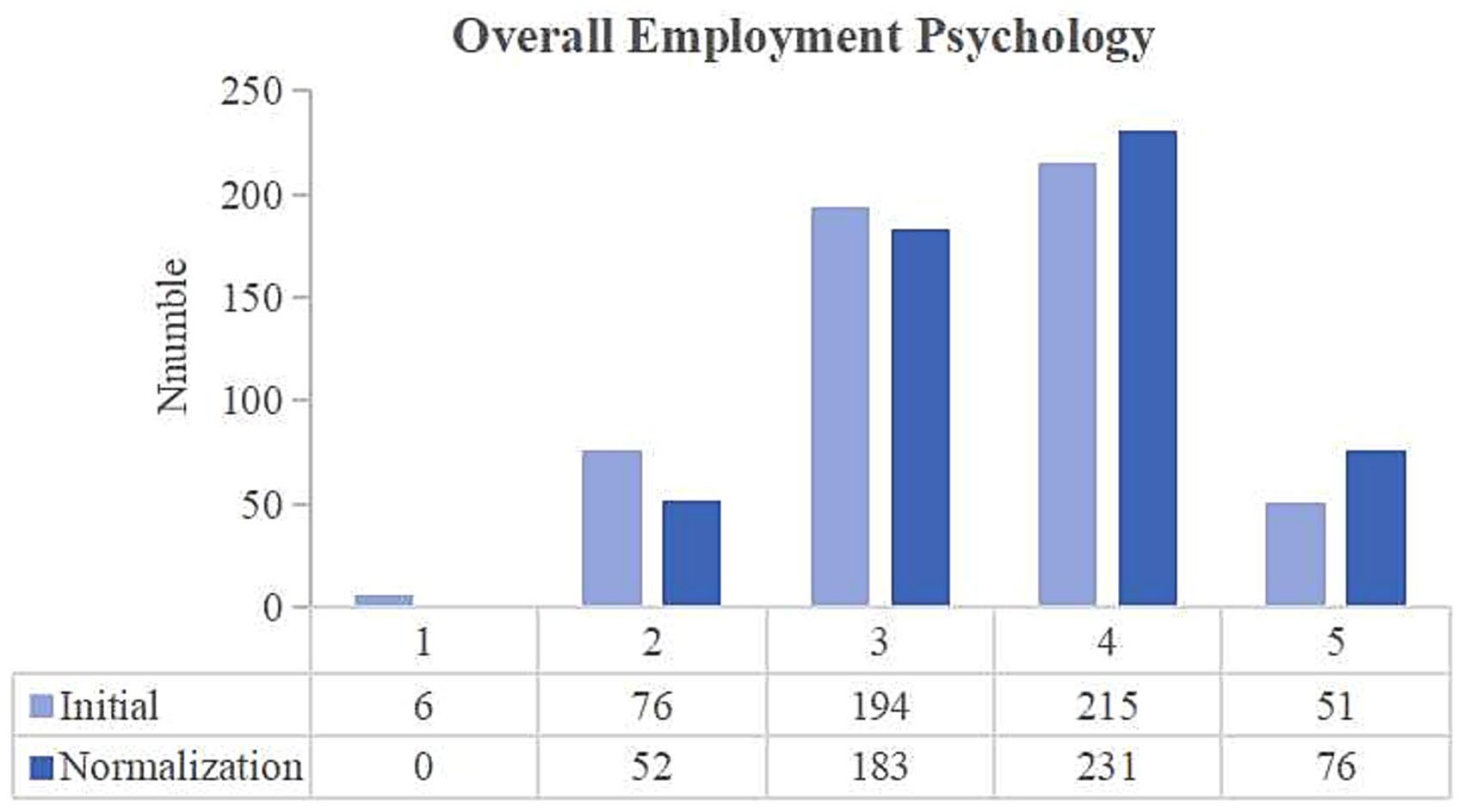
Changes in the overall employment psychology of university students between the two stages of COVID-19 pandemic control.

We used the snowball sampling method. Starting with a few eligible university students and expanding through them to more survey respondents until the required sample is completed. A total of 804 questionnaires were collected, 542 valid ones were obtained after excluding those from first year junior college and undergraduate students, as well as those with incomplete and careless responses. The article used SPSS statistical software to analyze the reliability and validity of the questionnaire. The Cronbach’s alpha was 0.845, which exceeded 0.7, the Kaiser-Meyer-Olkin was 0.834, and the Bartlett’s test of sphericity was *p* = 0.000, which was less than 0.05. Therefore, the questionnaire passed the tests of reliability and validity (Figures 1–6 and Table 1).

**Table 1:** Basic information of samples.

Variable	Category	Number	Percentage
Gender	Male	270	49.8
	Female	272	50.2
Age	20 years old and below	210	38.7
	21–25 years old	298	55.0
	26 years old and above	34	6.3
Household registration	Agricultural household	311	57.4
	Non-agricultural household	231	42.6
Marriage	Unmarried	496	91.5
	Married	46	8.5
Number of siblings	None	198	36.5
	One	179	33.0
	Two	124	22.9
	Three or more	41	7.6
Education	Junior college students	49	9.0
	Second grade	44	
	Third grade	5	
	Undergraduate students	373	68.8
	Second grade	171	
	Third grade	116	
	Fourth grade	86	
	Professional master’s students	69	12,7
	First grade	36	
	Second grade	19	
	Third grade	14	
	Academic master’s students	40	7,4
	First grade	12	
	Second grade	16	
	Third grade	12	
	Doctoral students	11	2.0
	First grade	7	
	Second grade	1	
	Third grade and above	3	
Major	Science and engineering	205	37.8
	Social sciences	79	14.6
	Economics and management	181	33.4
	Education	26	4.8
	Medicine	9	1.7
	Agriculture and forestry	26	4.8
	Arts and sports	16	3.0
Work experience	No work experience	365	67.3
	More than six months of work experience	177	32.7
Region	Northeast China	7	1.3
	North China	56	10.3
	East China	333	61.4
	Central China	55	10.1
	South China	29	5.4
	Southwest China	49	9.0
	Northwest	13	2.4
	Total	542	100.0

This paper collected 542 questionnaires from 170 universities in China. The samples showed more diverse characteristics in gender, age, household registration, marriage, number of siblings, education, major, work experience and region. The multidimensional sample structure ensures that our findings comprehensively reflect the employment psychology of Chinese university students in different contexts.

### 3.2 Changes in employment psychology of university students in the initial and normalization stages of pandemic control

#### 3.2.1 Career planning confusion psychology

The degree of confusion among university students was compared between the two stages of COVID-19 prevention and control. During the normalization stage, the number of respondents choosing scores of 4 and 5 increased by 41. In addition to the professional master’s students, there was an increase in the number of junior college, undergraduate, academic master’s and doctoral students choosing for scores of 4 and 5. Table 2 presents the results of the paired t-test which show a significant difference between the initial and normalization stages scores (*p*-value = 0.011, less than 0.05), thereby suggesting an increase in the severity of university students’ career planning confusion psychology. Such change may be explained by the prolonged pandemic control in China and the social isolation of these students, which made them feel confused about their future direction. This negative mood can easily spread to their employment by affecting their confidence and motivation to find a job after graduation.

**Table 2:** Paired sample t-test.

Paired sample t-test								
	Pair Difference					t	Degree of freedom	Sig. Double tail
	Average value	Standard deviation	Mean standard error	Confidence Interval 95%				
				Lower limit	Upper limit			
Confusion Initial-Normalization	−0.135	1.235	0.053	−0.239	−0.030	−2.539	541	0.011^**^
Anxiety Initial-Normalization	−0.303	1.192	0.051	−0.403	−0.202	−5.911	541	0.000^***^
Stability Initial-Normalization	−0.101	1.158	0.050	−0.199	−0.004	−2.040	541	0.042^**^
Uneasiness Initial-Normalization	−0.264	1.225	0.053	−0.367	−1.161	−5.016	541	0.000^***^
Inferiority Initial-Normalization	−0.081	1.239	0.053	−0.186	0.023	−1.525	541	0.128
Overall employment psychology Initial-Normalization	−0.884	3.948	0.170	−1.217	−0.551	−5.212	541	0.000^***^

*p < 0.05; **p < 0.01; ***p < 0.001.

**Question 1:** In the initial stage of COVID-19 control, indicate the degree to which you feel confused about your employment.

**Question 2:** In the normalization stage of COVID-19 control, indicate the degree to which you feel confused about your employment.

#### 3.2.2 Employment competition anxiety psychology

The degree of anxiety felt by university students as a result of their classmates finding employment was then compared. In the normalization stage, the number of respondents choosing the scores of 4 and 5 increased by 56. In addition to the professional master’s students, there was an increase in the number of junior college, undergraduate, academic master’s and doctoral students choosing for scores of 4 and 5. Table 2 presents the paired t-test results, which indicate a significant difference between the initial and normalization stages scores (*p*-value = 0.000, less than 0.01), thereby suggesting an increase in the number of university students who felt anxious after their classmates found employment. According to social comparison theory, individuals always try to compare themselves with their peers as a way of assessing their abilities and status (Festinger, 1954; Gerber et al., 2018). The COVID-19 pandemic considerably affected the operations of many enterprises, and drastically reduced the demand for labor in many small and medium-sized enterprises, thereby resulting in layoffs, recruitment suspensions, and reduced employment opportunities. During the normalization stage, despite China experiencing a gradual economic recovery, its employment situation remains fragile, and the competition for jobs remains fierce. When university students see their classmates succeeding in the job market (e.g., obtaining their ideal job, a high-paying position, etc.), they unconsciously make comparisons. Such comparisons may make them feel that they are lagging behind others, thus creating anxiety.

**Question 3:** In the initial stage of COVID-19 control, indicate the degree of anxiety you felt after your classmates already found employment.

**Question 4:** In the normalization stage of COVID-19 control, indicate the degree of anxiety you felt after your classmates already found employment.

#### 3.2.3 Employment stability preference psychology

The degree to which university students chose a particular job due to its stability was then compared between the two stages of COVID-19 prevention and control. In the normalization stage, the number of people who chose the score of 5 increased by 15. In addition to the junior college and undergraduate students, there was an increase in the number of professional master’s, academic master’s and doctoral students choosing for scores of 4 and 5. Table 2 presents the paired t-test results, which indicate a significant difference between the initial and normalization stages scores (*p*-value = 0.042, less than 0.05), thereby suggesting an increase in the number of university students who chose highly stable careers during the normalization stage. According to risk aversion theory, individuals tend to favor less risky options when faced with uncertainty. In the initial stage of COVID-19 control, many enterprises stopped their work and production, and many employees were subjected to pay cuts or layoffs. Only government positions were unaffected by these changes, thus potentially influencing university students’ perceptions of career choices. As a result, university students preferred stable jobs, such as government organizations, state-owned enterprises and public institutions, to avoid employment risks.

**Question 5:** In the initial stage of COVID-19 control, indicate the degree to which you chose this job because of its stability.

**Question 6:** In the normalization stage of COVID-19 control, indicate the degree to which you chose this job because of its stability.

#### 3.2.4 Professional prospect uneasiness psychology

The degree to which the university students felt uneasy because of the unknown career prospects of their majors was then compared. In the normalization stage, the number of respondents choosing the scores of 4 and 5 increased by 55. There was an increase in the number of junior college, undergraduate, professional master’s, academic master’s and doctoral students choosing for scores of 4 and 5. Table 2 presents the paired t-test results, which show a significant difference between the initial and normalization stages scores (*p*-value = 0.000, less than 0.01), thereby suggesting an increase in the number of university students who felt uneasy due to the unknown employment prospects of their majors. During the COVID-19 pandemic, some industries faced major difficulties and were forced to undergo major changes. As a result, university students became highly uncertain and insecure about the employment prospects of their majors.

**Question 7:** In the initial stage of COVID-19 control, indicate the degree to which you felt uneasy because of the unknown career prospects of your major.

**Question 8:** In the normalized stage of COVID-19 control, indicate the degree to which you felt uneasy because of the unknown career prospects of your major.

#### 3.2.5 Employability inferiority psychology

The degree to which university students felt inferior because of the mismatch between the high recruitment conditions and their abilities was then compared. In the normalization stage, the number of respondents choosing the scores of 4 and 5 increased by 18. An increase in the number of undergraduate and professional master’s students choosing for scores of 4 and 5, and a decrease in the number of junior college, academic master’s and doctoral students choosing for scores of 4 and 5. However, the paired t-test results in Table 2 show that the difference between the initial and normalization stages scores is not significant (*p*-value = 0.128, greater than 0.05), thereby suggesting that controlling the COVID-19 pandemic did not alleviate the low self-efficacy felt by university students. During the pandemic, these students were forced to take online courses and were given few opportunities to engage in extracurricular activities, such as social practice and internships, thus damping their confidence in their professional abilities. Prolonged social isolation may have also affected their self-efficacy, leaving them to doubt their abilities and values and develop an inferiority psychology.

**Question 9:** In the initial stage of COVID-19 control, indicate the degree to which you felt inferior because of the mismatch between the high recruitment conditions and your abilities.

**Question 10:** In the normalization stage of COVID-19 control, indicate the degree to which you felt inferior because of the mismatch between the high recruitment conditions and your abilities.

#### 3.2.6 Overall employment psychology

The above five employment psychologies of university students were combined into a new variable called “overall employment psychology,” which was scored from 5 to 25, with a higher the score corresponding to a more negative employment psychology. In the normalization stage, the number of respondents who chose the scores of 4 and 5 increased by 41. Table 2 presents the paired t-test results, which indicate a significant difference in the negative psychology of university students between the two stages (*p*-value = 0.000, less than 0.01), thereby indicating that these students’ employment psychology became more negative after the pandemic. The COVID-19 pandemic has a long-term potential impact on individuals (Su et al., 2023). In the normalization stage, the employment psychology of university students may still experience short-term deterioration due to the ‘after-effects’ of the pandemic, which will take some time to adjust (Tables 3–6).

**Table 3:** University students’ employment choices in the normalization stage.

Employment choices	Number	Percentage
Employment direction		
Participate in the work	221	40.8
The domestic further study	193	35.6
Go abroad/territory to study	90	16.6
Independent entrepreneurship	27	5.0
Others	11	2.0
Total	542	100
Employment organization		
Government organizations	53	24.0
Public institutions	71	32.1
State-owned enterprises	57	25.8
Private enterprises	21	9.5
Foreign-funded enterprises	15	6.8
Others	4	1.8
Total	221	100.0
Employment location		
Rural areas	11	2.0
Townships	33	6.1
Counties	79	14.6
First-tier cities	162	29.9
New first-tier cities	112	20.7
Second-tier cities	120	22.1
Third-tier and lower cities	25	4.6
Total	542	100.0
Willingness to return hometown for employment		
Very unwilling	82	15.1
Unwilling	309	57.0
Neutral	71	13.1
Willing	68	12.5
Very willing	12	2.2
Total	542	100.0
Perception of employment situation		
Very bad	222	40.9
Bad	103	19.0
Neutral	146	26.9
Optimistic	52	9.5
Very optimistic	19	3.5
Total	542	100.0

**Table 4:** Variable setting and description.

Variable	Variable description	Mean	Standard deviation
Explained variable			
Employment intention	Will you find employment or start a business directly after graduation? If no = 0, if yes = 1	0.46	0.499
Explanatory variable			
Employment psychology	5–25 points, the higher the score, the more negative the employment psychology of college students.	16.02	4.025
Control variable			
Gender	Male = 0, Female = 1	0.54	0.499
Age	20 years old and below = 1, 21–25 years old = 2, 26 years old and above = 3	1.68	0.588
Academic qualification	Junior college students = 1, undergraduate students = 2, professional master students = 3, master students = 4, doctoral students = 5	2.19	0.816
Major	Science and Engineering = 1, Social science = 2, Economics and management = 3, Education = 4, Medicine = 5, Agriculture and forestry = 6, Art and sports = 7	2.48	1.520
Marriage	Unmarried = 0, married = 1	0.07	0.255
one-child	Are you an one-child? If no = 0, if yes = 1	0.37	0.482

**Table 5:** Regression results.

Variable	Model 1	Model 2	Model 3
Employment psychology	0.048* (0.022)	0.049* (0.022)	0.057* (0.023)
Age (21–25 years old)	0.455* (0.187)	0.568** (0.190)	0.634** (0.217)
Age (26 years old and above)	1.197** (0.392)	1.895*** (0.461)	1.457** (0.551)
Gender (Female)	0.385* (0.178)	0.266 (0.182)	−0.035 (0.204)
one-child (yes)		−0.309 (0.189)	−0.395* (0.196)
Marriage (married)		−1.454*** (0.408)	−1.499*** (0.419)
Academic qualifications (undergraduate students)			−0.875** (0.336)
Academic qualifications (professional master students)			−0.358 (0.455)
Academic qualifications (master students)			−0.394 (0.502)
Academic qualifications (doctoral students)			0.430 (0.986)
Major (social sciences)			0.805** (0.309)
Major (economics and management)			0.712** (0.238)
Major (education)			0.187 (0.445)
Major (medicine)			−1.128 (0.843)
Major (agriculture and forestry)			0.603 (0.447)
Major (art and sports)			−0.278 (0.553)
Constant	−1.471*** (0.396)	−1.294** (0.403)	−0.952* (0.481)

*p < 0.05; **p < 0.01; ***p < 0.001. The variable takes the minimum value as the reference class.

**Table 6:** Regression results for the five psychologies.

	Model 4	Model 5	Model 6	Model 7	Model 8
Confusion	0.107 (0.081)				
Anxiety		0.134 (0.087)			
Stability			0.252** (0.090)		
Uneasiness				0.135 (0.083)	
Inferiority					0.148 (0.085)
Control variables	Control	Control	Control	Control	Control
Constant	−0.446 (0.416)	−0.526 (0.427)	−0.808 (0.421)	−0.542 (0.427)	−0.539 (0.416)

*p < 0.05; **p < 0.01; ***p < 0.001.

## 4 Influence of university students’ employment psychology on their employment intention in the post-pandemic era

### 4.1 University students’ employment choices in the normalization stage

#### 4.1.1 Employment direction

The results for Question 11 (“What is your employment plan after graduation”) show that those university students who plan to pursue further studies outnumber those who plan to work directly. The number of students in China pursuing graduate studies has gradually increased in recent years, with some university graduates pursuing further studies despite their initial plans to find employment directly after graduation. As a source of psychological comfort, these students often refer to the behavioral responses of people around them when making their employment decisions (Liu and Zhang, 2021). However, this herd mentality may limit the diversity of their employment choices.

#### 4.1.2 Employment organization

The results for Question 12 (“What type of organization do you want to work for the most”) show that university students are eager to work for institutions, state-owned enterprises, and party and government organizations due to their stability, good benefits, and high social prestige. This choice also reflects one of their main strategies to cope with market risks and economic uncertainty, that is, to pursue institutional employment as a safe haven from risks (Li, 2023b).

#### 4.1.3 Employment location

The results for Question 13 (“Where do you want to work”) indicate that university students prefer working in first-tier cities, such as Beijing, Shanghai, and Guangzhou. A significant number of these students chose to work in new first-tier and second-tier cities and counties, while only few expressed their willingness to work in third-tier and lower cities, townships, and rural areas. Economically developed cities have obvious advantages in terms of employment opportunities, wage levels, living conditions and personal development opportunities, thus attracting many university graduates.

#### 4.1.4 Willingness to return to hometown for employment

The results for Question 14 (“Are you willing to return to your hometown after graduation for work”) suggest that the low willingness of university graduates to return to their hometowns may be driven by the limited employment opportunities, restricted space for personal development, lack of social resources, poor quality of life, and absence of welfare benefits in their hometown.

#### 4.1.5 Perception of employment situation

The results for Question 15 (“What do you think about the employment situation in China after the pandemic prevention and control”), show the relatively pessimistic attitudes of university graduates. They generally perceived the post-pandemic employment situation as difficult.

### 4.2 Influence of the employment psychology of university students on their employment intention

#### 4.2.1 Research hypothesis

Stress interaction theory suggests that when stressor in the external environment act on the internal psychology of individuals, which affects the cognitive evaluation and coping behavior of individuals through interaction (Lazarus and Folkman, 1987). Different individuals will produce different types of cognitive appraisals when faced with a stressor, thus resulting in different coping styles and outcomes. When individuals perceive that stressors bring more gains than losses or when they have sufficient ability and resources to cope with these stressors, they tend to conduct challenging stress appraisals and usually adopt positive coping strategies. Otherwise, they tend to conduct hindering stress appraisals and choose negative coping strategies.

Macro-level economic and social factors (e.g., increase in unemployment rates and intensified competition for employment due to economic slowdown and the COVID-19 pandemic) and their impact on the labor market are the main drivers of changes in university students’ employment intentions and choices (Li, 2023b). According to stress interaction theory, the severe employment situation produces a sense of stimulation among university students that becomes a significant source of employment pressure. Other factors, such as the long-standing education model, family expectations, and peer pressure, also bring negative employment psychologies to university students, such as confusion, anxiety, and uneasiness. However, not all stressors have a negative impact on individuals (Li and Li, 2013), and a certain degree of positive stressors can stimulate them to engage in highly aggressive behaviors to cope with challenges (Sun and Yuan, 2023). Under such circumstances, university students may work harder to find employment opportunities and take the initiative to adapt to changes in the market, thereby turning pressure into motivation.

Public life course theory of intergenerational sociology suggests that encountering major socioeconomic changes or significant historical events during the critical period of adolescent growth can significantly affect young people’s values and behaviors and subsequently lead to the formation of intergenerational commonalities among youth groups (Li, 2020b). This theory also helps explain the current changes in university students’ employment intentions and choices. While transitioning from their schools to the labor market, the current generation of university students have encountered major historical events, such as China’s economic transformation and the COVID-19 pandemic, thus resulting in obvious group convergences in their propensity for employment intentions and choices (Li, 2023b). In the post-pandemic era, the overall employment psychology of these students became increasingly negative. To alleviate employment pressures and negativities, these students may show a greater willingness to find employment. Based on these arguments, the following hypotheses are proposed:

H1: In the post-pandemic era, the more negative the overall employment psychology of university students, the stronger their desire for employment.

Career planning confusion psychology: The COVID-19 pandemic profoundly affected the global job market and intensified the uncertainty and difficulty faced by university students in finding employment (Li et al., 2024). These students have no experience of coping with sudden shocks, and often feel confused and lost when planning their future career and development. Under this psychological state, these students may regard employment as a solution, that is, they may prioritize finding employment over choosing a career. When these students begin to understand their own interests and abilities after accumulating some work experience, they can choose a career development direction that suits them and gradually escape from their psychological state of confusion.

H1a: In the post-pandemic era, the stronger the career planning confusion psychology of university students, the higher their desire for employment.

Employment competition anxiety psychology: University students tend to compare their employment situation with that of their peers or classmates as a benchmark. When their peers find employment, university students feel some kinds of pressure that intensified their emotions and employment anxiety. Under such circumstances, these students may become increasingly desperate to find a job to alleviate their anxieties, reduce their pressure of competing and comparing themselves with their peers, and gain social recognition and a sense of self-worth.

H1b: In the post-pandemic era, the stronger the employment competition anxiety psychology of university students, the higher their desire for employment.

Employment stability preference psychology: The uncertainty in future economic returns will negatively affect the risk appetite of crisis-affected individuals, which in turn cuts down their willingness to take risks (Ahsan, 2014). Due to the impact of the COVID-19 pandemic, university students become worried about their future employment prospects and may lower their expectations of finding highly competitive and challenging jobs in favor of stable jobs with good benefits to secure their income. Individuals from disadvantaged socioeconomic backgrounds, value their need for economic survival more than their personal interests, dreams, or career ambitions (Carey et al., 2023). Due to the increased uncertainty during the COVID-19 pandemic, many university students favored stability above everything else and made highly conservative career choices (Liu and Zhang, 2021). In other words, these students actively search for stable jobs to avoid risks.

H1c: In the post-pandemic era, the stronger the employment stability preference psychology of university students, the higher their desire for employment.

Professional prospect uneasiness psychology: After the COVID-19 pandemic, those industries related to the majors pursued by university students may have undergone significant changes, thus introducing a feeling of uneasiness among university students about the employment prospects of their majors. Such uneasiness stimulates their sense of competition and prompts them to work harder to improve their employment competitiveness. To cope with the challenges brought about by industry changes, these students actively adjust their employment strategies and look for employment opportunities that suit them. Consequently, a stronger uneasiness about professional employment prospects corresponds to a stronger willingness of university students to find employment.

H1d: In the post-pandemic era, the stronger the professional prospect uneasiness psychology of university students, the higher their desire for employment.

Employability inferiority psychology: After the normalization of pandemic prevention and control, university students still held negative attitudes toward the present employment situation, which they generally perceived as “difficult.” Confidence is an important psychological factor influencing these students’ career choices (Wan and Liu, 2024). In the face of a severe employment situation, university students tend to lose their confidence and doubt their own ability, which becomes an internal pressure. However, when university students realize their lack of abilities, thus creating internal pressure. However, such internal pressure can also be regarded as a kind of incentive and motivation that prompts these students to work hard and improve their professional skills. By accumulating some work experience, these students can gradually overcome their low self-esteem and build their self-confidence.

H1e: In the post-pandemic era, the stronger the employability inferiority psychology of university students, the higher their desire for employment.

#### 4.2.2 Variable setting

1. Explained variable: employment intention. The question, “What is your future employment direction,” was designed to understand the influence of employment psychology and direct employment willingness of Chinese university students after their graduation. The options “participation in work” and “independent entrepreneurship” were combined into the new variable “employment intention,” while the options of “further study in China,” “study abroad,” and “other” were merged.
2. Explanatory variable: employment psychology. The five psychological problems faced by university students in their employment were combined into a single variable to reflect the overall negative employment psychology of these students. This variable was scored from 5 to 25, with a higher score corresponding to a more negative employment psychology. Employment psychology was divided into employment psychology in the initial stage and employment psychology in the normalization stage. Only the employment psychology of university students in the normalization stage of COVID-19 pandemic control was used as an explanatory variable in the regression.

#### 4.2.3 Analysis of regression results

The binary variable indicating whether university students are directly employed or start their own business after graduation was analyzed using the following binary logistic model:


$$\mathrm{Logit}\left(p\right)=Ln\left(p/1-p\right)=\beta_{0}+\beta_{1}\mathrm{psychology}+\overset{n}{\underset{i=1}{\sum }}\alpha_{i}x_{i}$$

where *β*_0_ is a constant term, *psychology* denotes the employment psychology of university students. *β*_1_ denotes the corresponding influence coefficient, X_i_ is a control variable, αis the corresponding influence coefficient of the control variable, and *p*/1 − *p* is the probability for university students to participate in work, engage in independent entrepreneurship, or pursue other employment directions after graduation.

##### 4.2.3.1 Overall analysis

To guarantee the robustness of the regression results, Model 1, 2, and 3 were obtained by adding the variables one by one. In Model 1, only the individual characteristics (gender and age) and employment psychology of university students were included. Based on this model, the family characteristics (marital status, and one-child) of these students were added to Model 2, and their educational characteristics (academic level and professional category) were added to Model 3.

1. Core variables (Models 1, 2, and 3): The university students’ employment psychology passed the significance test, indicating thay these students’ employment psychology positively and significantly affects their employment intention. In other words, the more negative the employment psychology of university students, the stronger their willingness to participate in work or start their own business after graduation. Based on stress interaction theory and public life course theory, under this influence mechanism, college student groups gradually form a self-directed mindset when facing employment challenges. Under certain circumstances, the negative employment mentality of college students can stimulate greater enthusiasm for job search and entrepreneurship.
2. Control variables (Model 3): **Age**. Age has a positive and significant effect on university students’ employment intention. Younger university students may choose to study at home, study abroad or not to be employed for the time being after graduation, while older university students are anxious to find a job to support their families. **Gender**. Gender has no significant effect on university students’ employment intention. **One-child**. One-child (yes) exerts a negative and significant effect. In other words, those university students who are not the only child in their families have a stronger willingness to work, while those students coming from one-child households can receive more financial and resource support from their parents and are thereby not in a hurry to find a job, or are willing to spend more time to find a satisfactory job. **Marriage**. Marriage (married) has a negative and significant effect on university students’ employment intention, indicating that unmarried university students have a stronger willingness to work, because they have less family constraints and can devote more time and energy to their work. Meanwhile, married university students who choose to return to their families may be prevented from entering the workforce by several factors, such as raising their children and taking care of their families. The higher anxiety among unmarried students than among married students may be explained by the economic downturn and poor employment situation in the country. **Academic qualifications**. Academic qualifications (college and undergraduate students) have a significant effect on university students’ employment intention, with undergraduate students experiencing a negative and significant effect. Compared with college students, undergraduate students are 0.4 times less willing to be employed. These undergraduates also have higher job and salary requirements, and if they do not find a satisfactory job for the time being, they may choose to remain unemployed to prepare for examinations for graduate school, the editorial board, or the public office. **Major**. Major (science and engineering, social sciences, economics and management) has a positive and significant effect on university students’ employment intention. Compared with science and engineering university students, social sciences and economics and management university students are 2.2 and 2.0 times more likely to be employed. The popularity of different majors in the job market varies and is influenced by the present economic situation and industry prospects.

##### 4.2.3.2 Analysis of regression results for the five psychologies

Regression analyses were conducted on the university students’ confusion, anxiety, stability preference, uneasiness, and inferiority psychology to empirically test the influence of these employment psychologies on their employment decisions after graduation. The control variables include age, gender, one-child, marriage, academic qualifications, and major.

The regression results for Model 4–8 show that only the employment stability preference psychology is significant at the 1% level. This psychology positively and significantly affects the employment intentions of university students. Individuals tend to favor less risky options when faced with uncertainty. Therefore, under the influence of the COVID-19, university students are governed by their stability preference psychology and are eager to find a secure job to avoid risks.

##### 4.2.3.3 Heterogeneity analysis of academic qualifications

The above analysis shows that the employment stability preference psychology positively and significantly affects the employment intentions of university students. Therefore, it is necessary to conduct a heterogeneity analysis of impact of the stability preference psychology on university students’ employment intentions in different educational background. Due to the small sample size of doctoral degree students, only 11 people, they were merged with the sample of master’s degree students. The regression results (Table 7) show that the stability preference psychology of junior college students is significant at the 1% level. This psychology positively and significantly affects the employment intentions of junior college students. The stability preference psychology of academic master’s degree (or above) is negative and significant at the 5% level. This psychology negatively and significantly affects the employment intentions of academic master’s students (or above). Our explanation is that highly educated students do not have concerns about employment and instead choose more challenging and high paying careers.

**Table 7:** Results of heterogeneity analysis of academic qualifications.

Variable	University students’ employment intention			
	Junior college students	Undergraduate students	Professional master’s students	Academic master’s and doctoral students
Employment stability preference psychology	1.292** (0.497)	0.194 (0.109)	0.436 (0.316)	−1.373* (0.666)
Control variables	Control	Control	Control	Control
Observation value	49	373	69	51

*p < 0.05; **p < 0.01; ***p < 0.001.

## 5 Conclusions and countermeasures

### 5.1 Conclusion

1. Chinese university students demonstrated stronger feelings of confusion, anxiety, stability, uneasiness, and inferiority during the normalization stage of COVID-19 prevention and control than the initial stage, thus leading to a more negative employment psychology.
2. During the normalization stage, these students held a highly pessimistic outlook on their employment situation and showed a preference for direct employment, particularly in public institutions, state-owned enterprises, and government organizations. They are also particularly interested in working in first-tier cities and are unwilling to seek employment in their hometowns.
3. The employment psychology of university students during the post-pandemic period significantly influenced their employment intentions. Age (20 years old and below, 21–25 years, 26 years old and above) and major (science and engineering, social sciences, economics and management) had positive and significant effects on their employment intentions, marriage (married), one-child (yes), and academic qualifications (junior college students and undergraduate students) exerted negative and significant effects, and gender produced an insignificant effect.
4. Among the five employment psychologies, only stability preference psychology exerted a positive and significant effect on the employment intentions of university students, especially students with junior college degrees, indicating that under the influence of COVID-19 pandemic, these students were governed by their psychology of seeking stability and showed their eagerness to find a stable job to avoid risks. In addition, employment stability preference psychology exerted a negative and significant effect on the employment intentions of highly educated students (academic master’s students or above), because they do not have concerns about employment and instead choose more challenging and high paying careers.

### 5.2 Countermeasures

1. **Government**: In view of the anxious mentality of university students during the stage of COVID-19 prevention and control normalization, the government should introduce perfect policies to protect these students’ employment at the macro level, offer employment assistance, create a fair employment environment, and stimulate these students’ confidence and enthusiasm in finding employment.
2. **School**: The university students’ employment psychology has a significant impact on their employment intention. Therefore, schools should not only adjust their training programs with the times, but also work on their students’ employment guidance and social practice to improve their vocational literacy and entrepreneurial ability. In this way, university students entering the job market are equipped with real skills. Schools should also offer psychological counseling to their students, set up special counseling institutions, and recruit full-time counseling staff.
3. **Enterprise**: The employment stability preference psychology plays a key role in shaping the employment intentions of university students. Therefore, enterprises should create more jobs that are conducive to the development of these students’ wisdom and expertise, improve the quantity and quality of their job supply, and improve their social security and welfare benefits.
4. **Family**: Parents should not exert too much employment pressure on their children, respect their employment choices, and give them additional support and encouragement. They should also observe their children’s emotional changes and communicate with them in time to help them alleviate their negative emotions and build their self-confidence.
5. **Individual**: University students should prioritize developing their knowledge and skills in order to maintain a good mindset and reduce their negative employment psychology even when facing major emergencies. They should also stablish a correct concept of employment and actively participate in social practice activities to accumulate work experience.

### 5.3 Research outlook

First, given that this study was based on online questionnaire data, some limitations may be observed in the sample selection. Second, the mental health status of the participating university students was self-reported and somewhat subjective. Future studies should consider improving the method and representativeness of their sample selection. Interviews and medical reports may also be used to investigate the employment mental health status of university students. The changes in the employment psychology of university students in the post-pandemic era may also be tracked in the long term.

## Data Availability

The original contributions presented in the study are included in the article, further inquiries can be directed to the corresponding author.

## References

[ref1] Ahsan A. D. (2014). Does natural disaster influence people’s risk preference and trust? An experiment from cyclone prone coast of Bangladesh. Int. J. Disaster Risk Reduct. 9, 48–57. doi: 10.1016/j.ijdrr.2014.02.005

[ref2] Brooks S. K. Webster R. K. Smith L. E. Woodland L. E. Wessely L. Greenberg N.. (2020). The psychological impact of quarantine and how to reduce it: rapid review of the evidence. Lancet 395, 912–920. doi: 10.1016/S0140-6736(20)30460-8, PMID: 32112714 PMC7158942

[ref3] Carey R. L. Bailey M. J. Polanco C. I. (2023). How the COVID-19 pandemic shaped adolescents’ future orientations: insights from a global scoping review. Curr. Opin. Psychol. 53, –101655. doi: 10.1016/j.copsyc.2023.101655, PMID: 37540938

[ref4] Feng J. Li X. Liu Q. (2022). Changes in employment status of graduates from top Chinese universities amidst the pandemic: based on employment quality reports from 8 universities (2017-2021). Univ. Educ. Sci. 6, 87–97. doi: 10.3969/j.issn.1672-0717.2022.06.10

[ref5] Festinger L. (1954). A theory of social comparison process. Hum. Relat. 7, 117–140. doi: 10.1177/001872675400700202

[ref6] Gerber J. P. Ladd W. Jerry S. (2018). A social comparison theory meta-analysis 60+ years on. Psychol. Bull. 2, 177–197. doi: 10.1037/bul0000127 29144145

[ref7] Griffin L. Riley R. (2022). Exploring the psychological impact of working during COVID-19 on medical and nursing students: a qualitative study. BMJ Open 12:e055804. doi: 10.1136/bmjopen-2021-055804, PMID: 35738645 PMC9226460

[ref8] Huang L. (2020). Employment research on college graduates during the COVID-19 pandemic: an empirical study based in Guangdong. Youth J. 3, 85–95. doi: 10.3969/j.issn.2095-7947.2020.03.013

[ref9] Kasperson R. Thomas W. Bonnie R. Jeannette S. (2022). The social amplification of risk framework: new perspectives. Risk Anal. 42, 1367–1380. doi: 10.1111/risa.13926, PMID: 35861634 PMC10360138

[ref10] Kelsey R. W. Ashley M. Joyce E. D. (2024). The psychosocial impact of the COVID-19 pandemic on adolescent and young adult Cancer survivors in the United States: an integrative review. J. Adolesc. Young Adult Oncol. 13, 80–96. doi: 10.1089/JAYAO.2023.0072 37797223

[ref11] Kira A. I. Shuwiekh A. H. Ashby S. J. Elwakeel A. S. Alhuwailah A. Sous F. M.. (2021). The impact of COVID-19 traumatic stressors on mental health: is COVID-19 a new trauma type. Int. J. Ment. Heal. Addict. 21, 1–20. doi: 10.1007/S11469-021-00577-0 PMC8259553 34248442

[ref12] Kumar B. D. Kofi F. A. Maria S. S. (2020). Impact of COVID-19 on psychology among the university students. Global Chall. 4:2000038. doi: 10.1002/gch2.202000038 PMC7537036 33042575

[ref13] Lazarus S. R. Folkman S. (1987). Transactional theory and research on emotions and coping. Eur. J. Personal. 1, 141–169. doi: 10.1002/per.2410010304, PMID: 39205987

[ref14] Lerner R. M. (2002). Concepts and theories of human development. 3rd Edn. London: Lawrence Erlbaum Associates.

[ref15] Lerner R. M. (2006). “Developmental science, developmental systems, and contemporary theories of human development” in Handbook of child psychology. Vol. 1: theoretical models of human development. eds. Damon W. Lerner R. M. Lerner R. M.. 6th ed (Hoboken, NJ: Wiley). pp. 1–17, 43–61, 542–548

[ref16] Li C. L. (2020b). Intergenerational sociology: a unique perspective on understanding the values and behavior patterns of China’s new generation. China Youth Res. 11, 36–42. doi: 10.3969/j.issn.1002-9931.2020.11.005

[ref17] Li C. L. (2020a). College student employment under pandemic impact: employment pressure, psychological stress, and changes in employment choices. Educ. Res. 41, 4–16.

[ref18] Li C. (2023a). A brief discussion on the mindset of university students in employment and its relief in the post-new crown epidemic context. Appl. Educ. Psychol. 4, 20–27. doi: 10.23977/APPEP.2023.040204

[ref19] Li C. L. (2023b). Changes in college students’ employment choices amidst increasing risk and competition. China Youth Soc. Sci. 42, 19–29. doi: 10.16034/j.cnki.10-1318/c.2023.05.011

[ref20] Li H. Dong G. Deng Y. Xie F. (2024). Discrepancy between employment expectations and realities among university students in the post-pandemic era: psychological adaptation. Curr. Learn. Explor. 2, 52–54. doi: 10.18686/cle.v2i1.3536

[ref21] Li J. Huang Y. Li Y. Luo A. (2021). Analysis of mental health status and influencing factors of fresh graduates during the COVID-19 pandemic. Mod. Hosp. 21, 464–469. doi: 10.3969/j.issn.1671-332X.2021.03.041 (in Chinese)

[ref22] Li Z. Li R. (2013). Review of challenge-stress and hindrance-stress sources. Foreign Econ. Manag. 35, 40–59. doi: 10.16538/j.cnki.fem.2013.05.008

[ref23] Li X. Xiang O. Gui Y. (2021). Between materialism and post-materialism: changes in college students’ attitudes towards employment in the post-pandemic era. Cult. Rev. 1, 120–159. doi: 10.3969/j.issn.1674-4608.2021.01.014

[ref24] Liu C. Zhang Y. (2021). Institutional entanglement: the impact of pandemic risk on college students’ employment values. Jianghan Acad. 40, 5–13. doi: 10.16388/j.cnki.cn42-1843/c.2021.04.001

[ref25] Park J. H. Khanh B. (2023). Mental health of undergraduates one year after the start of the COVID-19 pandemic: findings from the national university health assessment III. J. Am. Univ. Health 1, 1–4. doi: 10.1080/07448481.2022.2161822

[ref26] Priya B. Kakoli D. (2023). Status of mental health among university and university students during first and second wave of COVID-19 outbreak in India: a cross-sectional study. J. Affective Disord. Rep. 12:100494. doi: 10.1016/j.jadr.2023.100494, PMID: 36777966 PMC9894831

[ref27] Regnoli M. G. Rosa D. B. Palombo P. (2022). “Voice to the youth”: an interpretative phenomenological analysis of how Italian young adults experienced the pandemic. Mediterranean J. Clin. Psychol. 10, 1–30. doi: 10.13129/2282-1619/mjcp-3397

[ref28] Rodolfo R. Valentina S. Benedetto T. J. Giulia D. Sonia M. Francesca P.. (2023). Changes in mental health outcomes in the general population 14 months into the COVID-19 pandemic in Italy. J. Affect. Disord. 325, 35–40. doi: 10.1016/J.JAD.2022.12.148 36608856 PMC9810378

[ref29] Sae W. Taro Y. Akira S. (2022). Impact of the COVID-19 pandemic on the physical and psychological health of female university students in Japan. Nurs. Health Sci. 24, 634–642. doi: 10.1111/nhs.12962, PMID: 35656780 PMC9347817

[ref30] Su S.-Z. Gong Y.-M. Zhao Y.-M. Ni S.-Y. Shi L. Bao Y.-P.. (2023). Challenges of and responses to mental health problems in the post-COVID-19 era. J. Sichuan Univ. (Med. Ed.) 2, 217–222. doi: 10.12182/20230260301 PMC10409176 36949675

[ref31] Sun H. Yuan L. (2023). Research on the impact of new-generation employee status threat on elderly employees’ innovation behavior: based on stress interaction theory and impression management model. China Human Resour. Sci. 11, 68–80.

[ref32] Wan J. Liu F. (2024). Analysis of the psychological factors faced by the final year university students of China during job interviews and while choosing careers. J. Psycholinguist. Res. 53:24. doi: 10.1007/s10936-024-10045-0, PMID: 38446244

[ref33] Wang Z. Jiang B. Wang X. Niu Y. Xue H. (2022). Cross-sectional investigation and correlation analysis of psychology of university students returning to campus after COVID-19 lockdown lift. Front. Psych. 13:915042. doi: 10.3389/fpsyt.2022.915042, PMID: 35935405 PMC9352858

[ref34] Xu C. Wang X. Zou Y. (2023). Exploration of university students’ psychological problems based on online education under COVID-19. Psychol. Sch. 60, 3716–3737. doi: 10.1002/pits.22955

[ref35] Ye C. Chen Z. (2023). Analysis on the employment situation of college students and the path of accurate service guidance in the post-epidemic period. Modern Commerce Ind. 44, 98–99. doi: 10.19311/j.cnki.1672-3198.2023.21.033

[ref36] Yi C. (2024). The impact of mental health and the COVID-19 pandemic on employability and learning outcomes: evidence from Taiwanese university students. Discov. Sustain. 5:216. doi: 10.1007/S43621-024-00444-7

[ref37] Yu Y. Cui Y. (2021). Research on psychological counseling for college students’ employment in the post-pandemic period: a perspective based on psychological capital. Shanxi Youth 22, 164–165.

[ref38] Zhang L. (2023). Visual analysis of the trend of mental health changes among Chinese university students in the context of the normalisation of the new crown epidemic. Appl. Math. Nonlin. Sci. 8, 1093–1104. doi: 10.2478/amns.2021.2.00288

[ref39] Zhang X. Bian L. (2024). Influence of the first wave of COVID-19 on Chinese students’ psychology and behavior: a case study approach. Front. Psych. 15:1382301. doi: 10.3389/fpsyt.2024.1382301, PMID: 38957735 PMC11217512

[ref40] Zhang Q. Tan M. Zuo H. (2022). College students’ employment psychology and behavior under normalized pandemic prevention and control: a study based on the theory of planned behavior. Technol. Entrepreneurship Monthly 35, 117–120. doi: 10.3969/j.issn.1672-2272.202211152

[ref41] Zheng S. Wu G. Zhao J. Chen W. (2022). Impact of the COVID-19 epidemic anxiety on university students’ employment confidence and employment situation perception in China. Front. Psychol. 13:980634. doi: 10.3389/fpsyg.2022.980634, PMID: 36160584 PMC9501885

[ref42] Zhou L. Qi Y. Jiang J. (2023). The impact of COVID-19 on college students’ employment psychology and countermeasure suggestions: based on structural equation modeling. J. Hebei Univ. Sci. Technol. (Soc. Sci. Ed.) 23, 101–110. doi: 10.7535/j.issn.1671-1653.2023.01.013

